# Identification of a novel nonsense *SLC16A2* gene mutation in an infant with severe neurologic phenotype: A case report

**DOI:** 10.1097/MD.0000000000039047

**Published:** 2024-07-19

**Authors:** Wu Peng, Shuxia Shi, Liqi Yang, Deyun Liu

**Affiliations:** a Department of Pediatrics, The Second Affiliated Hospital of Anhui Medical University, Hefei, China; b Department of Clinical Medicine, Anhui Medical College, Hefei, China.

**Keywords:** Allan–Herndon–Dudley, MCT8, nonsense mutation, *SLC16A2* gene, thyroid hormone

## Abstract

**Rationale:**

Allan–Herndon–Dudley syndrome (AHDS) results from a pathogenic variant in the hemizygous subunit of the *SLC16A2* gene, which encodes monocarboxylate transporter 8 and follows an X-linked recessive pattern. AHDS manifests as neuropsychomotor developmental delay, intellectual disability, movement disorders, and thyroid hormone abnormalities. It is frequently misdiagnosed as cerebral palsy or hypothyroidism.

**Patient concerns:**

A 9-month-old male infant exhibited poor head control, hypodynamia, motor retardation, hypertonic limbs, and thyroid abnormalities. Despite levothyroxine supplementation and rehabilitation therapy, no improvements were observed. Whole-exome sequencing identified a novel nonsense mutation in *SLC16A2* (c.124G > T, p.E42X), which unequivocally established the diagnosis.

**Diagnoses:**

AHDS was confirmed.

**Interventions:**

Levothyroxine treatment commenced early in infancy, followed by 3 months of rehabilitation therapy, starting at 5 months of age. The combined administration of levothyroxine and methimazole was initiated at 1 year and 10 months of age, respectively.

**Outcomes:**

While improvements were noted in thyroid hormone levels, neurological developmental delays persisted.

**Lessons:**

AHDS should be considered in patients presenting with atypical neurological features and thyroid hormone abnormalities such as elevated triiodothyronine and decreased thyroxine levels. The early utilization of exome sequencing aids in prompt diagnosis. The identified *SLC16A2* nonsense mutation correlates with severe neurological phenotypes and adds to the spectrum of genetic variations associated with AHDS.

## 1. Introduction

Thyroid hormones (TH) play a pivotal role in nervous system development and their cellular entry is facilitated by transmembrane transporter proteins. Among these, monocarboxylate transporter 8 (MCT8), which belongs to the monocarboxylic transporter (MCT) family, is a crucial facilitator of TH.^[1]^ MCT8 is a protein composed of 539 amino acids encoded by *SLC16A2*, which spans 6 exons and 5 introns.^[2]^ Structurally, MCT8 comprises 12 transmembrane domains with intracellular amino-terminal and carboxy-terminal domains. Its expression is widespread across various tissues including the brain, heart, liver, kidney, intestine, placenta, and thyroid.^[3]^ It plays a key role in transporting the active forms of triiodothyronine (T3) and thyroxine (T4) across the blood-brain barrier into neurons.

Allan–Herndon–Dudley syndrome (AHDS; MIM 300523) is a rare X-linked recessive disorder that was first documented in 1944. It was later confirmed that after 60 years, mutations in the *SLC16A2* gene located in the Xq13.2 region of chromosome X.^[2]^ These mutations lead to MCT8 deficiency, hindering the transport of TH across the blood–brain barrier, resulting in inadequate neural TH supply. AHDS presents with a diverse and varied clinical spectrum and is characterized predominantly by neurological and endocrine symptoms. Evaluation of TH typically reveals elevated serum T3, decreased T4, and normal or slightly elevated thyroid-stimulating hormone (TSH) levels.^[1]^ Delayed myelination is a common finding on magnetic resonance imaging (MRI).^[4–6]^ Various types of mutations in *SLC16A2*, including nonsense, missense, insertion, frameshift, deletion, and splice site mutations, have been documented.^[7]^ These mutations lead to impaired protein synthesis, disrupted protein folding, transport anomalies, accelerated protein degradation, and the loss of thyroid hormone binding and transport. The severity of the AHDS clinical phenotype corresponded to the residual transport capacity of the mutated MCT8 protein.^[2,8]^ The type of mutation type may influence the residual transport capacity of MCT8, correlating with the severity of disease manifestations. Severe phenotypes are often associated with large deletions and frameshifts.^[9]^ Conversely, some single amino acid deletions and missense mutations result in only a mild reduction in MCT8’s ability to transport TH, leading to less severe neurodevelopmental outcomes.^[9]^ Nonsense mutations in *SLC16A2* are relatively rare. Here, we present a case of a newly identified nonsense mutation in an infant with AHDS that exhibited neurological impairments, such as hypodynamia, motor retardation, hypertonic limbs, and tremors. Additionally, we provide a summary of nonsense mutations reported to date.

## 2. Case report

This male Chinese infant was delivered via cesarean section at 39^+3^ weeks of gestation without neonatal asphyxia, with a birth weight of 3250 g. He was the second child of non-consanguineous healthy parents with no family history of endocrinological or neurological disorders. The patient’s older brother was in good health. Routine physical examinations at 3 months of age revealed failure to control his head. By five and a half months, he had been admitted to a pediatric neurological rehabilitation center due to poor head control. The patient displayed delayed responses to the auditory stimuli and exhibited minimal vocalizations. He was unable to roll over independently and demonstrated poor handgrip. Physical examination revealed weakness, motor retardation, and hypertonic limb weakness.

Given the observed neurodevelopmental delay, the first step in the assessment was thyroid hormone assay, considering their pivotal role in the nervous system. Results indicated serum T4 levels of 58.10 nmol/L (reference range 55.4–161.25), serum T3 levels of 4.33 nmol/L (reference range 1.01–2.96), serum free thyroxine levels of 8.87 pmol/L (reference range 10.44–24.38), serum free triiodothyronine levels of 10.70 pmol/L (reference range 2.77–6.31), and serum TSH levels of 5.331 mIU/L (reference range 0.38–4.34). Other metabolic indices, including blood lactate (1.53 mmol/L), blood ammonia (18 μmol/L), and blood homocysteine (8.38 μmol/L), were within normal limits. Due to the low free thyroxine levels, levothyroxine therapy was initiated.

**Figure 1.**
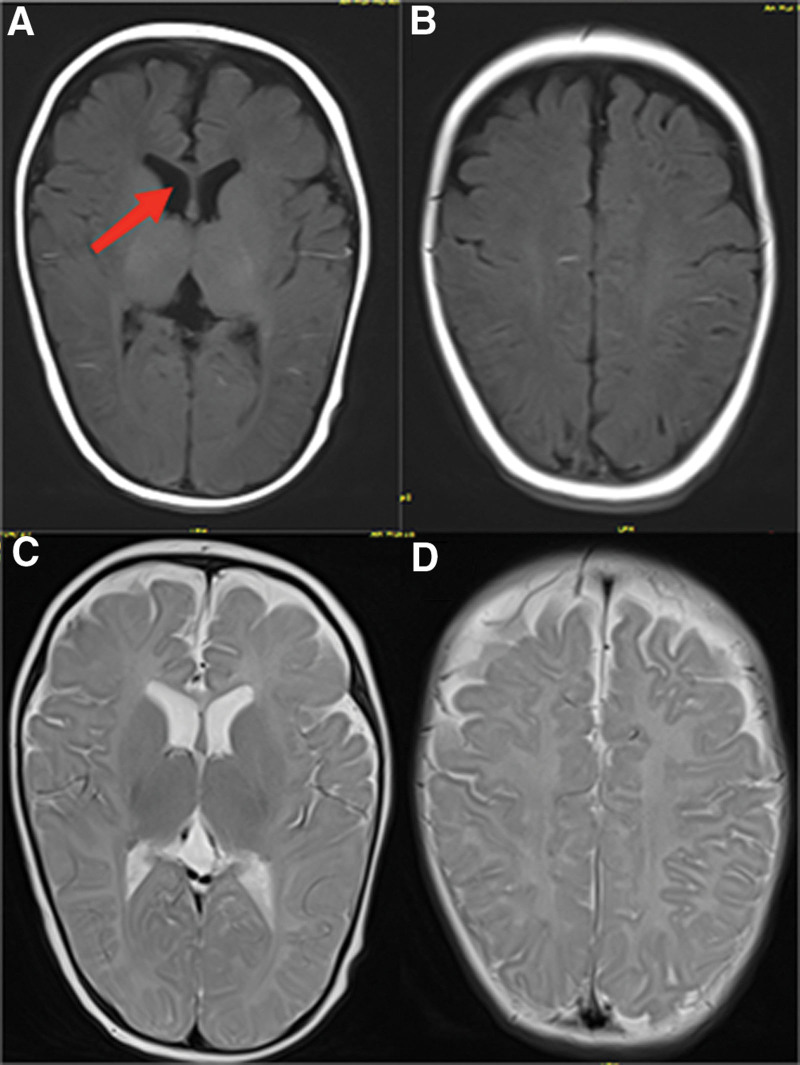
Brain MRI performed at five and a half months of age. Axial T1-weighted image shows no high signal in the knees of the corpus callosum, as represented by red arrows, which indicates delayed myelination. Decreased brain volume was observed simultaneously in T1-weighted (A and B) and T2-weighted images (C and D).

Brain MRI revealed bilateral frontotemporal subarachnoid widening, indicative of reduced brain volume, and unmyelinated knees of the corpus callosum, suggestive of delayed myelination (Fig. 1). The Gesell infant developmental assessment reported severe delays in coarse and fine motor development (developmental quotient, DQ = 38), moderate delays in object adaptation (DQ = 46), borderline verbal skills (DQ = 76), and mild delays in social adaptation (DQ = 63). A diagnosis of a global developmental disorder was established. Along with levothyroxine treatment, rehabilitation therapy, including relaxation training, joint mobilization, total body plyometrics, and unassisted gymnastics, was conducted for 3 months. However, no improvement in neurological manifestations was observed.

He was referred to our growth and development endocrinology clinic at the age of 9 months, 3 months after his initial presentation. Severe growth retardation persisted and was accompanied by an inability to control his head, roll over, or sit unaided. The patient exhibited spastic paralysis and was bedridden. Given the pattern of abnormal TH, notably elevated serum T3 and decreased T4 levels, along with motor dysfunction, dystonia, delayed myelination, and brain volume atrophy, suspicion arose regarding the association of the *SLC16A2* mutation with his condition.

**Figure 2.**
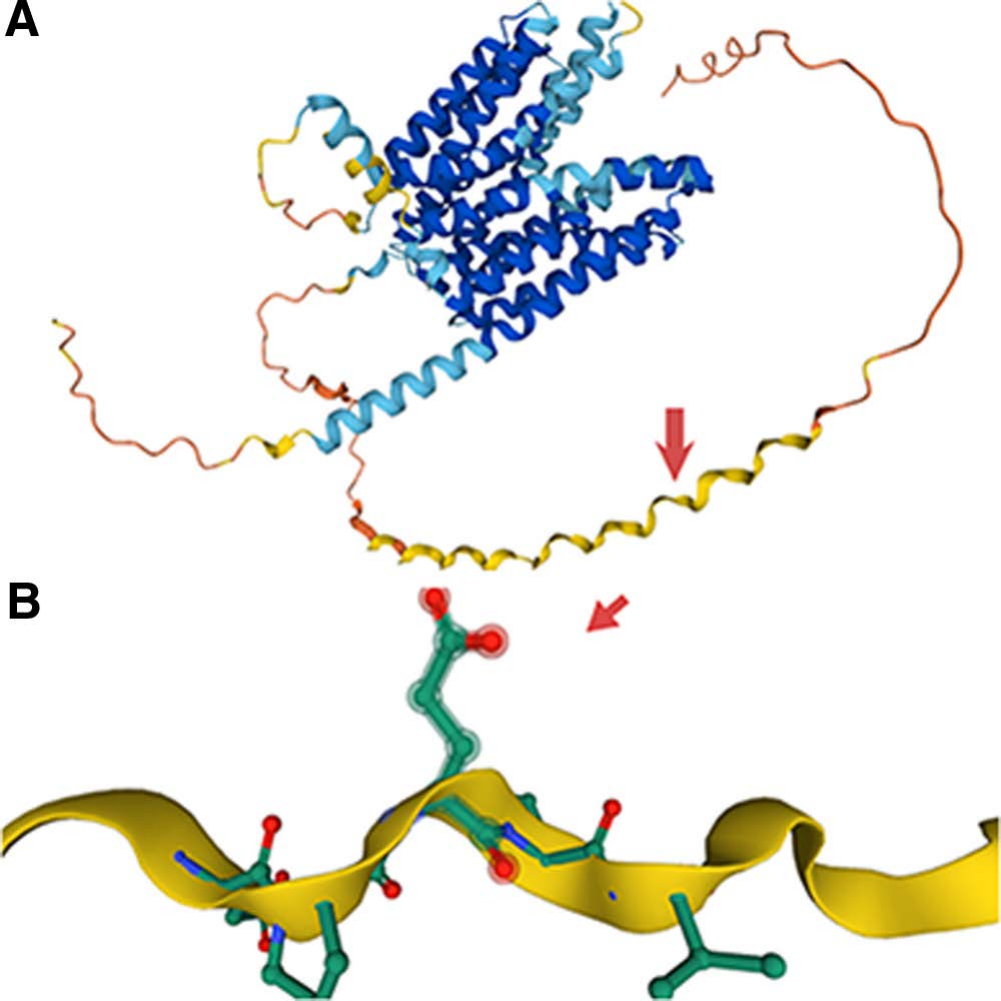
(A) Overview of the tertiary structure of the MCT8 protein. (B) Close-up of the amino acid side chain at NO.42 where coding is blocked due to the occurrence of the *SLC16A2* mutation c.124G > T, as represented by red arrows.

**Figure 3.**
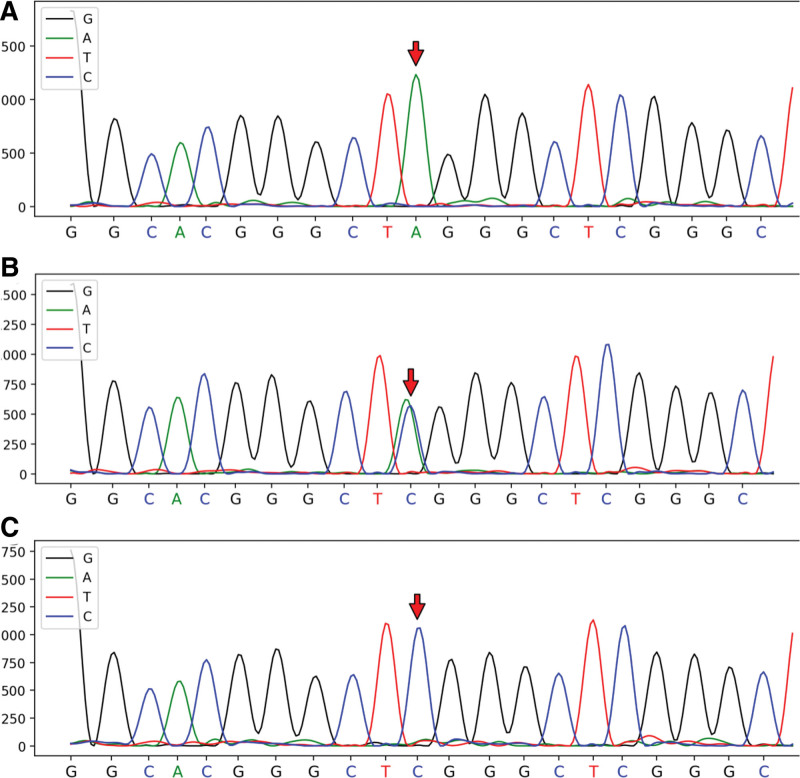
Chromatogram of the *SLC16A2* mutation: c.124G > T, chromosome position: chrX:73641596. (A) Sanger confirmation of the c.124G > T mutation in the proband. (B) Sanger sequencing of the heterozygous mother. (C) Sanger sequencing of the wild-type father.

**Figure 4.**
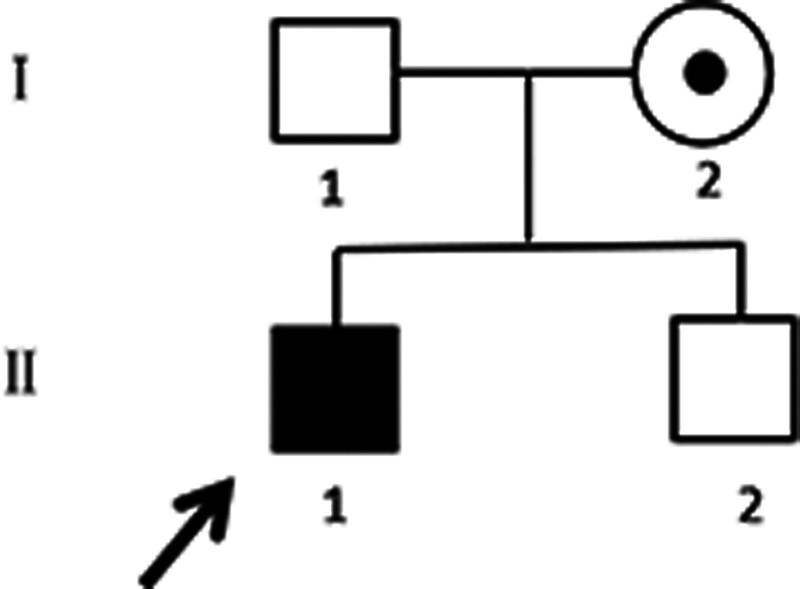
Pedigree of the family. The proband is shown with a black square, while the carrier mother is presented as a circle with black spot.

After obtaining parental consent, whole-exome sequencing analysis was conducted. This revealed a hemizygote nonsense mutation c.124G > T (p.E42X) in *SLC16A2*, located on chromosome X at chrX:73641596, transcript NM_006517, within exon 1. This mutation blocked MCT8 synthesis (Fig. 2). Subsequently, the patient’s mother was confirmed to be a heterozygous carrier of the mutation through Sanger sequencing for validation, consistent with an X-linked recessive inheritance pattern for the proband (Figs. 3 and 4).

The identified nonsense mutation in loss of function represents a significant mutation site in a gene with a known pathogenic principle (PVS). This specific mutation is not cataloged in the HGMD professional edition database, Clinvar database, population frequency data, or the gnomAD database (PM2) for the East Asian population. According to the ACMG guidelines, 2 pieces of evidence (PVS + PM2) support the likely pathogenic classification.

Owing to various considerations, the traditional combination of levothyroxine and methimazole was employed to reduce peripheral hyperthyroidism and improve central hypothyroidism in this patient. Despite improvements in the metabolic status and thyroid indicators, minimal progress in neurological development was observed. At 2 years of age, the patient weighed 11 kg and measured 84 cm long. He exhibited no conscious calls from his parents, made poor eye contact, and showed no recognition of people or objects. Walking or rolling over remains a challenge. Emerging symptoms included 1 or 2 tremors lasting approximately 30 minutes before bedtime, interfering with the patient’s sleep.

## 3. Discussion

Due to the absence of systematic epidemiologic investigations, the precise prevalence of AHDS remains unknown^[2]^ although it is believed to affect <1 in 1,000,000 individuals, predominantly males.^[8]^ A study by Li et al, utilizing exome sequencing in children diagnosed with “cerebral palsy,” identified the *SLC16A2* variant,^[10]^ suggesting potential cases of AHDS disguised as cerebral palsy. These patients might have received rehabilitation therapy without molecular confirmation of the diagnosis, as observed in the initial clinical experience in this case. Additionally, considering the number of reported cases in the literature, we hypothesized that this syndrome may be more common than previously thought.^[2]^

MCT8 has been identified as a specific active transporter for TH, which is crucial for T3 transport across the blood–brain barrier and T3 uptake into neuronal cells.^[11]^ Impaired thyroid hormone transport through the central nervous system during critical developmental stages, particularly in the first 2 years of life, can severely disrupt developmental processes and myelination,^[9]^ leading to neurological deficits such as mental retardation, dystonia, spastic paraplegia, central hypotonia, and delayed verbal development.^[8]^ Early manifestations often include generalized hypotonia, notably poor head and neck control, which is a primary concern during infancy. While axial hypotonia persists into adulthood, limb hypotonia progresses to spasticity and dystonia.^[11]^ Throughout childhood, cognitive impairments become increasingly apparent and are marked by delayed language acquisition and deficits in fundamental communication skills.^[12]^

Our patient exhibited early onset failure to control his head, hypodynamia, hypertonic limbs, motor retardation, absence of speech development, and a later onset of tremors. Poor head control, which is a notable symptom in early infancy, plays a crucial role in the diagnosis of AHDS. Achieving complete head control, indicative of improved neurodevelopment and longer survival, could serve as a relevant endpoint for future therapeutic trials targeting AHDS.^[12]^

The hallmark of this disorder is a distinctly abnormal thyroid hormone profile. Impaired T3 transport leads to the accumulation and elevation of typical peripheral T3 levels, whereas elevated free T3 inhibits TSH release, resulting in decreased free T4 levels.^[9]^ This interplay manifests as peripheral thyrotoxicosis and cerebral hypothyroidism.^[4,12]^ Elevated serum T3 levels are consistently observed in male patients with AHDS,^[13]^ and the associated presentation of elevated T3 and decreased T4 strongly indicates AHDS diagnosis. This patient exhibited the typical characteristics of thyroid hormone abnormalities, serving as a guide for follow-up exome sequencing. However, it is noteworthy that some AHDS cases with normal thyroid hormone levels have been reported, which are often associated with synonymous missense mutations.^[14]^

A crucial neurodevelopmental process regulated by TH is myelination, which follows a fixed temporal-spatial pattern.^[15]^ Delayed myelination, rather than hypomyelination, stands out as the most prominent MRI feature of AHDS^[6,15]^ potentially showing gradual improvement over time. Compared with normal infants of the same age, T1-weighted images of the corpus callosum knee in this patient, which typically exhibit high signal intensity indicating myelination, did not display such intensity, objectively suggesting delayed myelination. Additionally, a reduction in brain volume is commonly observed on MRI scans, including those of our patient.^[12,16]^ In a study by Kubota et al, delayed myelination was observed in 58.6% of patients, whereas brain atrophy was noted in 40.0% of patients.^[6]^ Nonetheless, these imaging findings are nonspecific and provide less diagnostic value for MCT8 disorders than thyroid hormone abnormalities. Ambulatory MRI monitoring may aid the identification of other white matter disorders. Furthermore, approximately 10% of patients may exhibit normal myelination patterns.^[17]^

The *SCL16A2* nonsense mutation c.124G > T (p.E42X) identified in this proband was inherited from his mother, who carried a heterozygous mutation. This mutation involves the replacement of guanine at position 124 with thymine in exon 1, resulting in the substitution of a glutamate codon with a premature termination codon at codon 42 of MCT8. The nonsense mutation is likely to undergo the nonsense-mediated mRNA decay pathway, in which mRNAs are degraded before translation into a polypeptide, ultimately leading to no protein production.^[5]^ This mutation, which has never been reported before, is predicted to be pathogenic. A search of the databases (Wanfang, CNKI, and PubMed) for studies published up to March 31, 2024, using the search terms “*SCLA16A2*,” “Allan–Herndon–Dudley,” “nonsense,” and “MCT8,” revealed 9 types of nonsense mutations in the *SCL16A2* gene resulting in AHDS (Table 1).^[5,13,18–23]^ Despite the absence of a predictive model for genotype-phenotype correlation, AHDS tends to be clinically severe when nonsense mutations cause structural deletion of MCT8 proteins or fail to produce MCT8, resulting in significant hypoplasia or loss of transporter T3 function. Among the reported cases, no mild cases were documented. The clinical manifestations include speech disorders, mental retardation, weakness, dystonia, dyskinesia, spasticity, and epilepsy. These cases were followed up for a short period, and the full extent of the clinical manifestations may be more severe in the long term. Typical thyroid hormone abnormalities were observed, except in 1 patient with normal TH assayed at 13 years of age.^[22]^ Delayed myelination was the most common MRI finding along with interstitial widening and cerebellar atrophy. Interestingly, there are similarities in the molecular pathogenesis of nonsense mutations and frameshift variants, both of which may lead to early termination codons and present with similarly severe clinical symptoms.^[7]^

**Table 1:** Summary of clinical reports of nonsense variants in *SCL16A2* gene.

Author, year and reference number	Nonsense mutation	Thyroid function	Brain MRI	Manifestation
Davide Tonduti (2013)^[5]^	c.656G > A[p.Trp219X]	FT3=, FT4↓, TSH=	Delayed myelination	Cognitive delay, dystonia, myoclonic seizures.
Jiaping Wang (2018)^[18]^	c.916C > T[p. Q306X]	T3↑, T4↓, TSH=	Delayed myelination	Mental retardation, hypotonia, dystonia.
Frints SG (2008)^[13]^	c.629insA[p.K210fsX241]	T3↑, T4↓, TSH=	NA	Dyskinesia, speech deficits, axial hypotonia, spasticity.
Jansen J (2007)^[19]^	c.733C > T[p.R245X]	T3↑, T4↓, TSH↑	NA	Axial hypotonia, spastic or flaccid quadriplegia, dystonic dyskinesia, and speech deficits.
Fen Lu (2022)^[20]^	c.1455delG[p.Val485Valfs*8]	T3↑, T4↓, TSH=	Diminution of brain volume	Spasticity, speech delay, movement disorders.
Fen Lu (2022)^[20]^	c.504_529del[p.Leu168Leufs*64]	T3↑, T4↓, TSH=	Diminution of brain volume	Spasticity, speech delay, dyskinesia, muscle weakness.
V Herzovich (2007)^[21]^	a single C→T[p.Q261X]	T3↑, T4↓, TSH=	Cerebellar atrophy	Mental retardation, lack of head control, axial hypotonia, spastic quadriplegia, sporadic dyskinesia, decreased muscle mass.
Tsurusaki Y (2011)^[22]^	c.1102A > T[p.R368X]	FT3=, FT4=, TSH=	Delayed myelination	Spasticity and dystonia with deep tendon hyperreflexia, as well as myoclonic and tonic seizures, tracheomalacia.
Cole J (2018)^[23]^	c.268C > T[p.Q90X]	T3↑, T4↓, TSH=	Decreased white matter	Spastic quadriplegia, trunk hypotonia, extensor posturing, generalized myoclonic epilepsy, severe cognitive impairment, intermittent inward strabismus, death.

**Abbreviations:** ↑, ↓,  =  = increased, decreased, normal, respectively; FT3 = free triiodothyronine, FT4 = free thyroxine. NA, not available.

The primary challenge in addressing AHDS is the alleviation of neuropathy.^[1]^ Presently, there are no approved therapies for treating AHDS. However, significant progress has been made in the development of thyroid hormone analogs over the past decade, with some currently undergoing clinical trials. TRIAC, a typical thyroid hormone analog, does not require passage through the cell membrane via MCT8. Its controlled thyrotropic activity holds the potential for restoring normal function to affected organs, presenting a promising avenue for treatment.^[1,24]^ Genetic counseling plays a crucial role in mitigating the risk of further cases within affected families. As an X-linked recessive disorder, mothers carrying the *SLC16A2* mutation have a 50% chance of passing the disease to their male offspring, while daughters have a 50% chance of inheriting the mutation and becoming carriers.

## 4. Conclusion

In summary, we present a case of *SCL16A2* nonsense mutation exhibiting a severe clinical phenotype characterized by global neurodevelopmental delay, with the patient generally failing to reach developmental milestones. This case underscores the importance of recognizing thyroid-specific alterations (elevated T3, decreased T4) alongside neurological delay and delayed myelination, necessitating a high index of suspicion for AHDS and advocating early exome sequencing. Our findings contribute to the expanding spectrum of nonsense mutations in *SCL16A2* and provide valuable new insights for genotype–phenotype correlation studies.

## Acknowledgments

The authors would like to thank the patient and his parents for allowing us to publish this case report.

## Author contributions

**Conceptualization:** Wu Peng, Shuxia Shi.

**Data curation:** Wu Peng, Shuxia Shi, Liqi Yang.

**Formal analysis:** Wu Peng, Deyun Liu.

**Investigation:** Wu Peng, Shuxia Shi.

**Methodology:** Liqi Yang.

**Supervision:** Wu Peng.

**Writing – original draft:** Wu Peng, Shuxia Shi.

**Writing – review & editing:** Wu Peng, Shuxia Shi, Liqi Yang, Deyun Liu.
